# Gait characteristics related to fall risk in patients with cerebral small vessel disease

**DOI:** 10.3389/fneur.2023.1166151

**Published:** 2023-06-06

**Authors:** Yajing Wang, Yanna Li, Shoufeng Liu, Peipei Liu, Zhizhong Zhu, Jialing Wu

**Affiliations:** ^1^Clinical College of Neurology, Neurosurgery and Neurorehabilitation, Tianjin Medical University, Tianjin, China; ^2^Department of Neurology, Tianjin Huanhu Hospital, Tianjin Key Laboratory of Cerebral Vascular and Neurodegenerative Diseases, Tianjin Neurosurgical Institute, Tianjin, China; ^3^Department of Rehabilitation, Tianjin Huanhu Hospital, Tianjin, China

**Keywords:** cerebral small vessel disease, walking, fall, gait analysis, gait parameters

## Abstract

**Background:**

Falls and gait disturbance are significant clinical manifestations of cerebral small vessel disease (CSVD). However, few relevant studies are reported at present. We aimed to investigate gait characteristics and fall risk in patients with CSVD.

**Methods:**

A total of 119 patients with CSVD admitted to the Department of Neurology at Tianjin Huanhu Hospital between 17 August 2018 and 7 November 2018 were enrolled in this study. All patients underwent cerebral magnetic resonance imaging scanning and a 2-min walking test using an OPAL wearable sensor and Mobility Lab software. Relevant data were collected using the gait analyzer test system to further analyze the time-space and kinematic parameters of gait. All patients were followed up, and univariate and multivariate logistic regression analyses were conducted to analyze the gait characteristics and relevant risk factors in patients with CSVD at an increased risk of falling.

**Results:**

All patients were grouped according to the presence or absence of falling and fear of falling and were divided into a high-fall risk group (*n* = 35) and a low-fall risk group (*n* = 72). Logistic multivariate regression analysis showed that the toe-off angle [odds ratio (OR) = 0.742, 95% confidence interval (CI) 0.584–0.942, *p* < 0.05], toe-off angle coefficient of variation (CV) (OR = 0.717, 95% CI: 0.535–0.962, *p* < 0.05), stride length CV (OR = 1.256, 95% CI: 1.017–1.552, *p* < 0.05), and terminal double support CV (OR = 1.735, 95% CI: 1.271–2.369, *p* < 0.05) were statistically significant (*p* < 0.05) and were independent risk factors for high-fall risk in patients with CSVD.

**Conclusion:**

CSVD patients with seemingly normal gait and ambulation independently still have a high risk of falling, and gait spatiotemporal-kinematic parameters, gait symmetry, and gait variability are important indicators to assess the high-fall risk. The decrease in toe-off angle, in particular, and an increase in related parameters of CV, can increase the fall risk of CSVD patients.

## 1. Introduction

Cerebral small vascular disease (CSVD) refers to a series of imaging and clinical manifestations that characterize a syndrome caused by any functional or structural pathological damage to small cerebral vessels, such as terminal arterioles, venules, and capillaries. Gait disorder is one of the main symptoms of CSVD patients (1, 2). Studies have shown that most falls of stroke patients after discharge occurs during walking, and gait disorder is an independent predictor of patients' fall risk. However, most studies are described by scale, observation, and other methods, with low accuracy, strong subjectivity, and limited dimensions (3). With the development of ergonomics, a large number of gait parameters can be obtained using wearable sensor devices, and gait symmetry and gait variability can be calculated (4). Both reflect the ability to maintain a stable and consistent walking rhythm in the motor control system. However, we found that there were few studies on gait symmetry and variability in patients with CSVD (5, 6). Therefore, we used wearable devices to measure the parameters related to the gait of patients with CSVD. Furthermore, we calculated gait symmetry and variability. We investigated the gait parameters that contributed to the high risk of falls among follow-up patients with a high risk of falling. Through our study, we can provide early rehabilitation treatment for patients with CSVD and reduce the occurrence of falls.

## 2. Materials and methods

### 2.1. Patients

In this study, 119 patients with CSVD were hospitalized at the Department of Neurology of Tianjin Huanhu Hospital, Tianjin, China between 17 August 2018 and 7 November 2018. The study was registered with the Chinese Clinical Trial Registry (Clinical Trial Registration No. ChiCTR2100042031) and was approved by the Ethics Committee of Tianjin Huanhu Hospital. All patients underwent head magnetic resonance imaging, including T2, fluid-attenuated inversion recovery, diffusion-weighted imaging, apparent diffusion coefficient, and gradient echo sequences. All patients were able to walk and complete the evaluation independently. All the clinical data were complete.

The inclusion criteria were as follows: (1) age ≥18 years; (2) met the diagnostic criteria for mild stroke [National Institutes of Health Stroke Scale (NIHSS) score ≤3]; (3) according to the pathogenesis, the selection (Trial of Org 10172 in Acute Stroke Treatment) type was small-artery occlusion; (4) all participants could walk independently and safely for 2 min without help from others or assistive devices; and (5) all participants were fully informed of the research process and signed the informed consent form.

The exclusion criteria were as follows: clinical diagnosis of dementia, mental disorders, severe cerebral hemorrhage, and other systemic diseases affecting gait, such as joint injury, arthritis, cervical spine disease, and lumbar spine disease.

**Figure 1:**
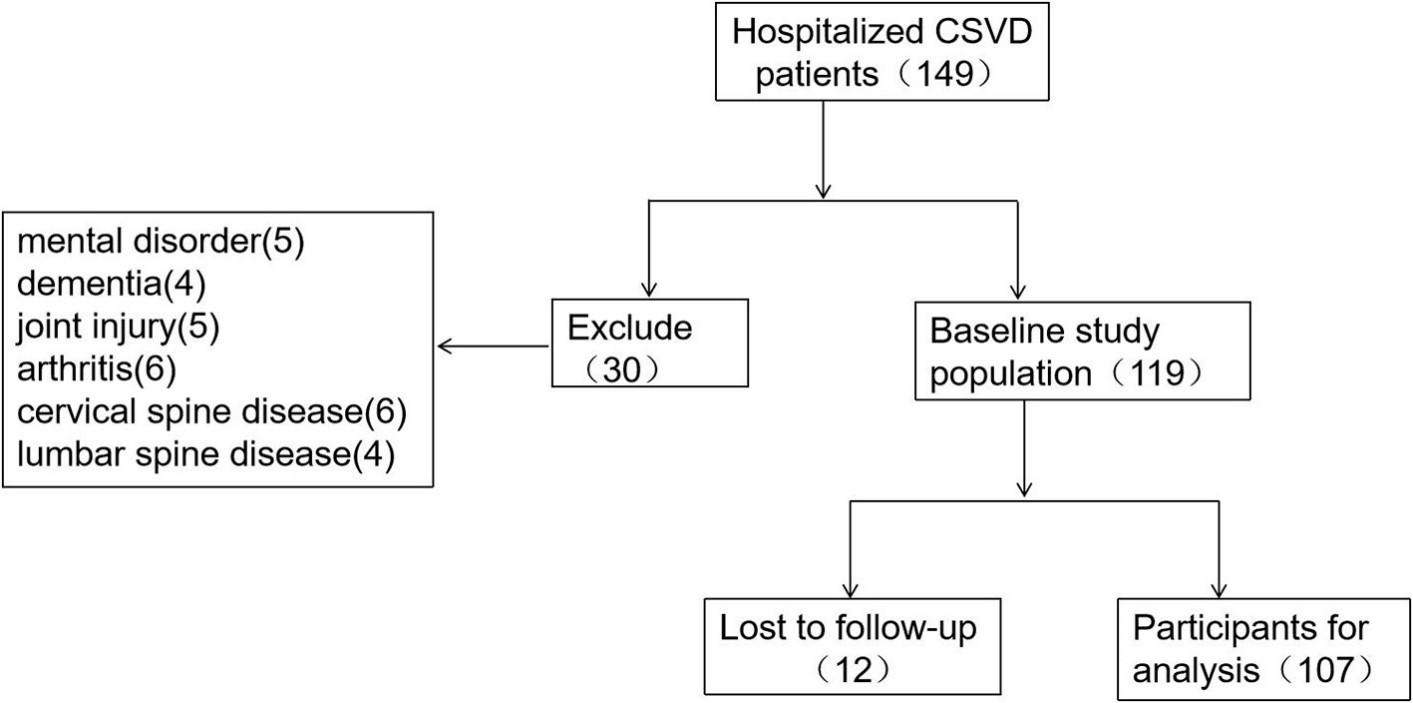
Flow chart of the inclusion and exclusion process of included patients.

Participants were selected according to inclusion criteria and exclusion criteria (Figure 1).

### 2.2. Study design

#### 2.2.1. General assessment

General clinical data of all patients, including age, sex, height, weight, and medical history, were collected and analyzed.

#### 2.2.2. Assessment of gait function

All enrolled patients underwent a 2-min walk test under the guidance of a physician, which was conducted in an empty room dedicated to the evaluation. The patients walked freely along a straight line which could be turned back for 2 min. Patients were simulated in advance to ensure that they were familiar with the test. At the start of the experiment, the OPAL wearable sensor (APDM) and Mobility Lab software (https://apdm.com/wearable-sensors/) were used for the 2-min walking test. The instrument had a total of six sensors, which were placed on the patient's body by professionals according to the following positions. The first sensor was worn at the uppermost sternal handle of the sternum, the second sensor was worn at the lowermost fifth lumbar vertebra at the lumbosacral junction, and the third and fourth sensors were worn on the dorsal side of the bilateral wrist joint. The fifth and sixth sensors were worn on the dorsum of both feet.

#### 2.2.3. Assessment of gait parameters

Gait parameter data were collected through the gait analysis and test system and transmitted to a computer terminal. Then, the time-space and kinematic gait parameters were analyzed. Gait symmetry was assessed using the Asymmetry Index (AI). The AI was calculated as follows:


$$\begin{array}{l} \text{AI}=|\text{XL}-\text{XR}|/\text{max}\left(\text{XL},\text{XR}\right)\times \;100, \end{array}$$

with L and R representing the left and right sides of the patient, respectively (5). X represents the corresponding gait parameter used in the analysis. This study mainly included stride length, single-limb support, terminal double support, swing, foot strike angle, and toe-off angle. Gait variability was assessed using the coefficient of variation. First, the coefficient of variation (CV) was calculated using the formula “standard deviation/mean,” which represents the gait variability (CVL represents the left variability and CVR represents the right variability). The next step was to integrate the variability of the left and right gait parameters using the formula (7, 8):


$$\begin{array}{l} \sqrt{\left(CVL+CVR\right)/2}\times 100. \end{array}$$

The parameters included were stride length, single limb support, terminal double support, swing, toe-off angle, and foot strike angle.

#### 2.2.4. Main outcomes

All patients were followed up for the presence of falls and the presence of fear of falling (FOF). The assessment of FOF was as follows: According to the FOF scale developed by American scholar Tinetti in 1993 (9), patients were asked, “Are you afraid or worried about falling?” The presence of FOF was determined by answering “not afraid,” “slightly afraid,”' “somewhat afraid,” and “very afraid.” Those who answered “not afraid” were defined as having no FOF, and those who answered with any other option were defined as having a FOF. Based on the presence of FOF or falling, the patients were divided into a high-fall risk (HFR) group and a low-fall risk (LFR) group. If the patient had no history of falls and answered as “not afraid,” they were assigned to the LFR group, and patients who had a history of falls or answered “slightly afraid,” “somewhat afraid,” or “very afraid,” were assigned to the HFR group.

### 2.3. Statistical analysis

Data processing was performed using SPSS 24.0 software (SPSS Inc., Chicago, IL, USA). Normally distributed numerical data were presented as the mean ± standard deviation (x ± s), and the independent sample *t*-test was used for comparison between the groups. Numerical data that showed a skewed distribution were presented as the median (the first quartile, third quartile) [M (Q1, Q3)], and the Mann–Whitney *U*-test was used for comparison between groups. Count data were expressed in the form of cases (percentage) [*n* (%)], and an *X*^2^ test was used for comparison between groups. Data from both groups were analyzed using multivariate logistic regression, and a *p*-value of <0.05 was considered statistically significant. In this study, logistic regression was used for the multivariate analysis of the two groups of data.

## 3. Results

### 3.1. Baseline clinical characteristics

The demographic characteristics of the LFR and HFR groups are summarized in Table 1. A total of 119 patients were included in this study, and the mean (SD) age was 59.55 (9.89) years. In total, 94 cases were male patients, 25 cases were female patients, and 12 cases were lost to follow-up, including the LFR (72 cases) and HFR group (35 cases). Age, sex, height, weight, diabetes mellitus, cardiac disease, and smoking did not differ significantly between the groups; however, the presence of hypertension was higher in the HFR group compared with the LFR group (*p* < 0.05) (Table 1).

**Table 1:** Demographic characteristics of participants.

	**LFR group (*n* = 72)**	**HFR group (*n* = 35)**	***X*^2^/*t*-value**	***P*-value**
Male [*n* (%)]	54 (75.00)	28 (80.00)	0.329	0.566
Age (year)	59.28 ± 10.46	61.80 ± 7.54	−1.274	0.206
Height (cm)	168.96 ± 6.54	170.43 ± 7.37	−1.046	0.298
Weight (kg)	74.25 ± 12.94	73.37 ± 10.75	0.347	0.729
Hypertension [*n* (%)]	48 (67.6)	30 (85.7)	3.995	0.047^a^
Diabetes [*n* (%)]	26 (36.6)	13 (37.1)	0.003	0.958
cardiac disease [*n* (%)]	9 (12.7)	7 (20.0)	0.981	0.322
smoke [*n* (%)]	40 (56.3)	26 (74.3)	3.214	0.073

LFR group, low-fall risk group; HFR group, high-fall risk group.

a P < 0.05.

### 3.2. Gait analysis in the LFR group and HFR group

Compared to patients in the LFR group, patients in the HFR group had lower stride frequency, slower stride speed, shorter stride length, and longer gait cycle time (*p* < 0.05). Simultaneously, the proportion of double limb support and terminal double support increased during each gait cycle, while the proportion of single limb support and swing decreased (*p* < 0.05). As shown in Table 2, the patients in the HFR group had smaller foot strike and toe-off angles (*p* < 0.05) (Table 2).

**Table 2:** Comparison of time-space and kinematic parameters between the two groups (*x* ± s).

	**Mean** ±**SD**		***t*-value**	***P*-value**
	**LFR group (*****n*** = **72)**	**HFR group (*****n*** = **35)**		
Stride frequency (steps/min)	106.4 ± 9.21	100.20 ± 12.52	2.909	0.004^a^
Stride speed (m/s)	0.92 ± 0.21	0.79 ± 0.25	2.832	0.006^a^
Stride length (m)	1.03 ± 0.20	0.93 ± 0.23	2.386	0.019^a^
Gait cycle (s)	1.14 ± 0.10	1.22 ± 0.19	−3.030	0.003^a^
Double limbs support (%GCT)	22.50 ± 5.64	25.32 ± 7.16	−2.220	0.029^a^
Single limb support (%GCT)	38.75 ± 2.81	37.36 ± 3.58	2.199	0.030^a^
Terminal double support (%GCT)	11.24 ± 2.79	12.62 ± 3.57	−2.186	0.031^a^
Swing (%GCT)	38.76 ± 2.80	37.36 ± 3.59	2.202	0.030^a^
Foot strike angle (degrees)	18.48 ± 6.18	14.73 ± 6.64	2.877	0.005^a^
Toe off angle (degrees)	33.17 ± 5.17	29.45 ± 6.37	3.233	0.002^a^

LFR group, low-fall risk group; HFR group, high-fall risk group.

a P < 0.05.

### 3.3. Comparison of gait symmetry between the two groups

This study compared gait symmetry between the two groups and found that the foot strike angle AI increased in patients in the HFR group (*p* < 0.05), whereas the stride length AI, toe-off angle AI, single-limb support AI, terminal double support AI, and swing AI showed no significant differences between the groups (*p* > 0.05) (Table 3).

**Table 3:** Comparison of gait symmetry between the two groups [%, M (Q1, Q3)].

	**LFR group (*n* = 72)**	**HFR group (*n* = 35)**	***Z*-value**	***P*-value**
Stride length AI	0.96 (0.77, 1.69)	1.55 (0.77, 2.99)	−1.755	0.079
Single limb support AI	1.74 (0.77, 3.69)	2.77 (1.13, 5.16)	−1.454	0.146
Terminal double support AI	8.03 (2.94, 16.31)	10.66 (4.41, 19.96)	−1.627	0.104
Swing AI	1.72 (0.83, 3.82)	2.65 (1.15, 4.96)	−1.481	0.139
Foot strike angle AI	10.96 (5.84, 22.55)	19.89 (7.69, 33.91)	−2.105	0.035^a^
Toe off angle AI	4.46 (2.04, 6.57)	6.66 (3.68, 13.49)	−1.939	0.053

LFR group, low-fall risk group; HFR group, high-fall risk group; AI, asymmetry index.

a P < 0.05.

### 3.4. Comparison of gait variability between the two groups

After analyzing gait variability in both groups, we found that the stride length CV, single limb support CV, terminal double support CV, swing CV, foot strike angle CV, and toe-off angle CV were significantly higher in the HFR vs. LFR group (*p* < 0.05) (Table 4).

**Table 4:** Comparison of gait variability between the two groups [%, *M* (*Q*1, *Q*3)].

	**LFR group (*n* = 72)**	**HFR group (*n* = 35)**	***Z*-value**	***P*-value**
Stride length CV	20.67 (18.43, 25.64)	23.95 (19.93, 26.80)	−2.085	0.037^a^
Single limb support CV	16.10 (14.27, 18.94)	18.75 (16.25, 20.92)	−2.962	0.003^a^
Terminal double support CV	30.46 (28.16, 33.16)	33.22 (29.30, 37.50)	−3.054	0.002^a^
Swing CV	16.08 (14.13, 19.91)	18.67 (16.68, 22.08)	−3.114	0.002^a^
Foot strike angle CV	34.56 (28.75, 42.24)	40.34 (32.69, 53.28)	−2.626	0.009^a^
Toe off angle CV	22.18 (19.06, 25.22)	26.11 (24.06, 29.40)	−3.758	0.000^b^

LFR group, low-fall risk group; HFR group, High-fall risk group; CV, coefficient of variation.

a P < 0.05.

b P < 0.001.

### 3.5. The regression analysis of influencing factors

All statistically significant gait indicators were included in the logistic multivariate regression analysis. The parameters of the toe-off angle, toe-off angle CV, stride length CV, and terminal double support CV were statistically significant (*p* < 0.05). These were identified as independent risk factors for falling in patients with CSVD. However, the remaining time-space and kinematic parameters of gait, gait symmetry parameters, and gait variability parameters were not statistically significant (*p* > 0.05) (Table 5).

**Table 5:** Results of the multivariate logistic regression analysis.

	**β value**	**SE**	**wald**	***P*-value**	**OR value**	**95% CI**	
**Lower limit**						**Upper limit**
Stride frequency (steps/min)	−0.110	0.395	0.078	0.780	0.896	0.413	1.943
Stride speed (m/s)	0.122	0.179	0.466	0.495	1.130	0.795	1.606
Stride length (m)	−0.052	0.167	0.097	0.756	0.949	0.684	1.318
Gait cycle (s)	0.016	0.267	0.004	0.952	1.016	0.603	1.714
Double limbs support (%GCT)	−0.045	0.556	0.007	0.935	0.956	0.321	2.843
Single limb support (%GCT)	−0.293	1.100	0.071	0.790	0.746	0.086	6.445
Terminal double support (%GCT)	−1.035	1.022	1.024	0.312	0.355	0.048	2.636
Swing (%GCT)	−0.901	1.074	0.703	0.402	0.406	0.049	3.336
Foot strike angle (degrees)	0.084	0.130	0.424	0.515	1.088	0.844	1.403
Toe off angle (degrees)	−0.299	0.122	6.015	0.014^a^	0.742	0.584	0.942
Foot strike angle AI	0.023	0.024	0.864	0.353	1.023	0.975	1.073
Stride length CV	−0.332	0.150	4.911	0.027^a^	0.717	0.535	0.962
Foot strike angle CV	0.113	0.067	2.800	0.094	1.119	0.981	1.277
Toe off angle CV	0.228	0.108	4.496	0.034^a^	1.256	1.017	1.552
Single limb support CV	−0.348	0.367	0.899	0.343	0.706	0.344	1.449
Terminal double support CV	0.551	0.159	12.027	0.001^a^	1.735	1.271	2.369
Swing CV	−0.499	0.402	1.539	0.215	0.607	0.276	1.336
Hypertension	1.080	0.691	2.442	0.118	2.944	0.760	11.408

AI, asymmetry index; CV, coefficient of variation.

a P < 0.05.

## 4. Discussion

### 4.1. Fall and fear of falling in patients with CSVD

Falls caused by gait disorders in patients with CSVD seriously affect their quality of life and are closely related to a poor prognosis (1, 2). FOF is a precursor to falling in patients with CSVD. FOF refers to the reduction of confidence or fall efficacy to avoid falling while participating in certain activities that the patients are capable of (9). Some studies (10) have shown that FOF not only exists in elderly people who have a history of falling but also in elderly people who have never experienced a fall. FOF reduces the patients' confidence in activities, which is not conducive to the rehabilitation of motor function in patients with stroke and affects the recovery of their neurological function. One study has shown that the incidence of FOF in patients with stroke during hospitalization was 54%, and the incidence of FOF in stroke patients after discharge was 32–66% (11). Our study found that the incidence of FOF in patients with CSVD who could walk independently was 32.71%. The possible reasons are related to the included population in this study and the time from onset to follow-up. The mean age of CSVD patients included in this study was 59.55 ± 9.89 years, NIHSS score ≤3 points, and the time from onset to follow-up was 4 years. Therefore, younger patients, mild stroke, and short follow-up time may lead to a lower risk of falls.

### 4.2. Time-space parameters and kinematic parameters of gait in patients with CSVD

Spatio-temporal parameters and kinematic parameters are important indicators of gait. Abnormal spatio-temporal and kinematic parameters often lead to gait instability, and patients are more likely to fall (12). In our study, we found that patients with CSVD in the HFR group had a shortened stride length, reduced stride frequency, and decreased stride speed. A reduction in stride length usually means a reduction in forward propulsion force and impairment of balance (13). Stride speed reflects the movement ability of an individual, and a decrease of 0.1 m/s has a significant clinical significance (14). The decline in walking speed reflects a decrease in the propulsion of the gait and is a sign of gait damage, and patients are more likely to fall when they want to increase their walking speed or are not prepared. In our study, the average stride speed in the HFR group decreased by 0.13 m/s compared to the LFR group, i.e., fall risk increased. We found that not only was the gait cycle of patients in the HFR group extended but also the proportion of double limb support and terminal double support in the gait cycle was extended, and the proportion of single limb support was reduced. This was to prevent falls and maintain body balance, which further absorbed shocks and maintained load-bearing stability by extending the time both feet contact the ground during double limb support and shortening the instability of single limb support (15). Stride frequency is negatively correlated with the gait cycle (16). When the gait cycle is prolonged, the gait frequency decreases, which is a compensatory manifestation of gait. Once entering the decompensation stage, the probability of patients falling increases sharply. Swing mainly reflects an individual's floor-clearance ability (17). The proportion of swing-phase patients in the HFR group was significantly shortened, resulting in a reduction in their floor clearance ability.

We included the important parameters of gait kinematics: the foot strike angle and toe-off angle. The foot strike angle reflects shock absorption and maintains forward stability (18), while the toe-off angle reflects the forward gait force, which is an important reflection of the ground clearance ability of the foot (19). In our study, we found that the foot strike and toe-off angles in patients with high-fall risk decreased during walking, indicating that CSVD not only affects the stability of the body moving forward but also affects the driving force for forward movement, resulting in patients with decreased ground clearance ability of the foot, increased instability, and increased fall risk.

### 4.3. Symmetry and variability of gait in patients with CSVD

In our study, not only the conventional gait parameters were analyzed, but the symmetry and variability of gait were quantified using formulas. The AI of the foot strike angle in the HFR group was significantly higher compared to the LFR group. In related studies (20), human gait symmetry is typically assumed to be consistent with left and right gait functions. The gait symmetry of healthy humans can effectively reduce the energy consumption of walking, reduce the risk of falling, and provide a stable and comfortable walking mode (21). The higher the AI, the greater the asymmetry deviation of the bilateral limbs during walking, and the higher the influence on the stability of walking (22). Therefore, the results of our study indicated that the lower extremities of patients in the HFR group had greater differences in shock absorption and maintenance of progressive stability while walking stability was poor. Compared with the LFR group, the AI of each gait parameter in the HFR group was higher, indicating that the gait asymmetry of the left and right limbs was more prominent in the HFR group during walking. Some studies have found that the increase of gait parameter asymmetry in stroke patients during walking is related to the decrease in progressive stability, impaired balance function, and increased risk of falls. Our further analysis found that there was a significant difference in the AI elevation of the foot strike angle between the two groups, and the foot strike angle reflected the foot clearance ability, so our study showed that the foot clearance ability of patients in the HFR group increased asymmetrically and increased the risk of fall.

Gait variability refers to the stability of gait during walking, which reflects the ability of the motion control system to maintain a stable and consistent walking rhythm (23). However, some studies have shown that the heterogeneity of gait time-space parameters is large, and gait variability is more effective in evaluating fall risk (24). In our study, we found that the stride length CV, single-limb support CV, terminal double support CV, swing CV, foot strike angle CV, and toe-off angle CV of patients with CSVD in the HFR group were significantly higher than those in the LFR group, suggesting that patients in the HFR group had decreased gait stability during walking. In addition, increased gait variability leads to increased energy expenditure, resulting in difficulty in maintaining postural balance and a higher risk of falls (25). We further included the indicators with statistical differences between the two groups for logistic regression analysis and found that toe-off angle, toe-off angle CV, stride length CV, and terminal double support CV were independent risk factors for falls in patients with CSVD. Terminal double support, which accounts for 10% of the gait cycle, is mainly responsible for weight release and weight transfer during walking (26). Its increased variability indicates that as the inconsistency of weight release and transfer of the lower limbs increases, the ability of the body to maintain stability decreases, and the risk of falling increases.

## 5. Conclusion

The high risk of falls in patients with CSVD is closely related to the time-space and kinematic parameters of gait, as well as the symmetry and variability of gait. In particular, toe-off angle, toe-off angle CV, stride length CV, and terminal double support CV were independent risk factors for falling in patients with CSVD. Our study could promote a better understanding of the risk factors for falling caused by gait disturbance in patients with CSVD. Our findings provide evidence for clinical work, which is helpful in administering targeted drugs or early rehabilitation intervention. However, this study has some room for improvement. First, some kinematic parameters, such as knee joint and hip joint, could be included while selecting some kinematic parameters which have the greatest impact on gait. In this way, it would be more sufficient in reflecting the comprehensive effect of cerebral microvascular disease on gait. Second, we could conduct a multicenter study and carry out rehabilitation interventions on gait disorders in patients so as to provide a more effective basis for guiding clinical treatment.

## Data availability statement

The datasets presented in this study can be found in online repositories. The name of the repository and accession number can be found below: Figshare, https://figshare.com/, doi: 10.6084/m9.figshare.22096259.

## Ethics statement

The studies involving human participants were reviewed and approved by Ethics Committee member: Xinping Wang, Yanming Zhang, Jinling Zhang, Tong Han, Yi Li, Yangui Xu, and Mingyu Li. Chairman: Xinping Wang. The patients/participants provided their written informed consent to participate in this study.

## Author contributions

YW conceived the study and was responsible for all research aspects. YL collected the data. SL and PL completed all statistical analyses and wrote the manuscript. ZZ assessed the gait function of patients. JW reviewed and commented on the data analysis and drafts. All the authors critically reviewed and approved the final version of the manuscript.
